# MOG_35 − 55_-induced EAE model of optic nerve inflammation compared to MS, MOGAD and NMOSD related subtypes of human optic neuritis

**DOI:** 10.1186/s12974-025-03424-4

**Published:** 2025-04-07

**Authors:** Erin N. Capper, Edward F. Linton, Jeffrey J. Anders, Randy H. Kardon, Oliver W. Gramlich

**Affiliations:** 1 Department of Ophthalmology and Visual Sciences, University of Iowa, 200 Hawkins Drive, Iowa City, IA 52242 USA; 2 Center for the Prevention and Treatment of Visual Loss, Iowa City VA Health Care System, Iowa City, IA 52246 USA; 3 Department of Neuroscience and Pharmacology, University of Iowa, Iowa City, IA 52242 USA

**Keywords:** Multiple sclerosis, MS, Myelin oligodendrocyte glycoprotein antibody-associated disease, MOGAD, Neuromyelitis Optica spectrum disorder, NMOSD, Optic neuritis, EAE, Animal models

## Abstract

Optic neuritis (ON), or inflammation of the optic nerve, is a common presenting symptom of demyelinating neuroinflammatory conditions that result in significant, subacute vision loss. Given its association with visual impairment and varying extent of visual recovery, ON has been recognized as a significant health burden with a need for new therapeutic strategies to improve long-term visual outcomes. Among the resources utilized to study ON, animal models have emerged as powerful tools to examine the underlying pathophysiology and the effectiveness of proposed therapies. In the current review, we discuss the functional and structural phenotypes related to ON in currently used mouse models, and summarize how the pathophysiology and visual phenotype of the myelin oligodendrocyte glycoprotein 35–55 (MOG_35 − 55_) experimental autoimmune encephalomyelitis (EAE) mouse model recapitulates clinical features of multiple sclerosis (MS), myelin oligodendrocyte glycoprotein antibody-associated disease (MOGAD), and neuromyelitis optica spectrum disorder (NMOSD). The location of ON and the amount of visual recovery in the EAE model most closely resembles MS and NMOSD. However, we propose that the MOG_35 − 55_-induced EAE model of ON is primarily a MOGAD model given its similarity in pathophysiology, spinal cord demyelination pattern, and the degree of vision loss, retinal nerve fiber layer (RNFL) swelling, and disc edema. Overall, the MOG_35 − 55_-induced EAE animal model demonstrates overlapping features of autoimmune demyelinating conditions and serves as a comprehensive tool to further our understanding of visual impairment in all three conditions.

**Supplementary Information:**

The online version contains supplementary material available at 10.1186/s12974-025-03424-4.

## Background

Optic neuritis (ON) is an inflammatory, demyelinating disease of one or both optic nerves resulting in subacute vision loss. ON has many etiologies, usually associated with an underlying autoimmune demyelinating disorder such as multiple sclerosis (MS), myelin oligodendrocyte glycoprotein (MOG) antibody-associated disease (MOGAD), or neuromyelitis optica spectrum disorder (NMOSD) [1]. Given the propensity for ON and resulting visual impairment, demyelinating disorders have been recognized as a serious disability resulting in reduced quality of life [2, 3]. Thus, there is a critical need to focus on new therapeutic strategies to reduce vision loss and other neurological dysfunction in this population using relevant animal models. For this paper, an extensive literature review was conducted utilizing the PubMed and Google Scholar platforms. Specifically, we targeted articles in these databases using the following search terms: “optic neuritis”, “optic neuritis animal model”, “optic neuritis treatment”, “multiple sclerosis”, “MS”, “myelin oligodendrocyte glycoprotein antibody-associated disease”, “MOGAD”, “neuromyelitis optica spectrum disorder”, “NMOSD”, “experimental autoimmune encephalomyelitis” and “EAE”. Additional articles were also added if they suited the context of the discussion. In the first chapter of this review, we aim to summarize the functional and structural phenotypes of MS, MOGAD, and NMOSD ON. The second chapter summarizes the pathophysiology and visual phenotype of the MOG_35 − 55_ experimental autoimmune encephalomyelitis (EAE) mouse model, its alternative, and other refined in vivo models. Next, we discuss the translational value of the MOG_35 − 55_ EAE model of ON and its limitations, summarize our findings and identify and future directions.

## Clinical presentation of optic neuritis

### Distinct characteristics of optic neuritis in patients with MS, MOGAD, and NMOSD

The term ON refers to optic nerve inflammation caused by heterogeneous pathobiology that largely results in subacute vision loss with variable response to treatment, resulting in partial or complete visual recovery. Common symptoms of ON include retrobulbar pain exacerbated by eye movements, dyschromatopsia— reduced color perception, and central vision loss. Clinically, ON is most frequently the result of autoimmune- demyelination associated with MS, MOGAD, and NMOSD [1–4]. Other phenotypes of ON are rare complications of systemic disorders such as systemic lupus erythematosus and rheumatic diseases, or occur in association with infections (syphilis, herpes, measles, mumps, etc.) [5, 6], vaccinations, insect stings, cancer-associated autoimmune responses, and immune modulation with chemotherapeutic agents [7–12]. Around one-third of cases of ON remain idiopathic with no identifiable cause.

ON, typically unilateral, is the initial presenting symptom in ~ 25% of new MS cases, and 70% of MS patients experience at least one episode of ON during disease progression with a 35% chance of recurrence [13–15]. In general, recovery of vision is good in more than 90% of affected eyes, but patients experience a significant decrease in vision-related quality of life [14, 16]. Transient decreases in vision are often precipitated by an increase in core body temperature (Uthoff’s phenomena) and can also interfere with driving, reading, sports, and other visually demanding activities. Moreover, recovery of vision in MS patients with recurring, especially bilateral, ON is delayed and limited [17]. The rate of ON as the presenting symptom in NMOSD (35%) is similar to MS, whereas ON is a more common initial presentation of MOGAD (55%) [18, 19]. Compared to the unilateral ON frequently seen in MS, both MOGAD and NMOSD tend to have recurring bilateral ON, with more frequent episodes, and severe vision loss at nadir. MOGAD patients may have significant optic disc edema during acute ON, a finding that is often absent or mild in patients with MS and NMOSD [6]. With regards to final visual outcome, patients with MOGAD-ON and NMOSD-ON have worse visual acuity than those with MS-ON, with NMOSD-ON having the poorest outcome overall [3, 20]. Six to fourteen% of severe MOGAD-ON cases experience minimal visual recovery with vision of 20/200 or worse [21], and 60–69% of NMOSD-ON cases [19]. While limited data is available for MOGAD and NMOSD, research has shown that vision loss occurs in MS patients even without a history of ON; therefore, it is not surprising that visual impairment in MS is recognized as one of the most common disabling manifestations [22]. MS-ON, MOGAD-ON, and NMOSD-ON display similar magnitude of acute visual field loss and impairment of optic nerve conduction speed as measured by perimetry and visual evoked potential (VEP) recordings.

### Neuro-ophthalmic and radiologic assessments of optic neuritis

**Fig. 1:**
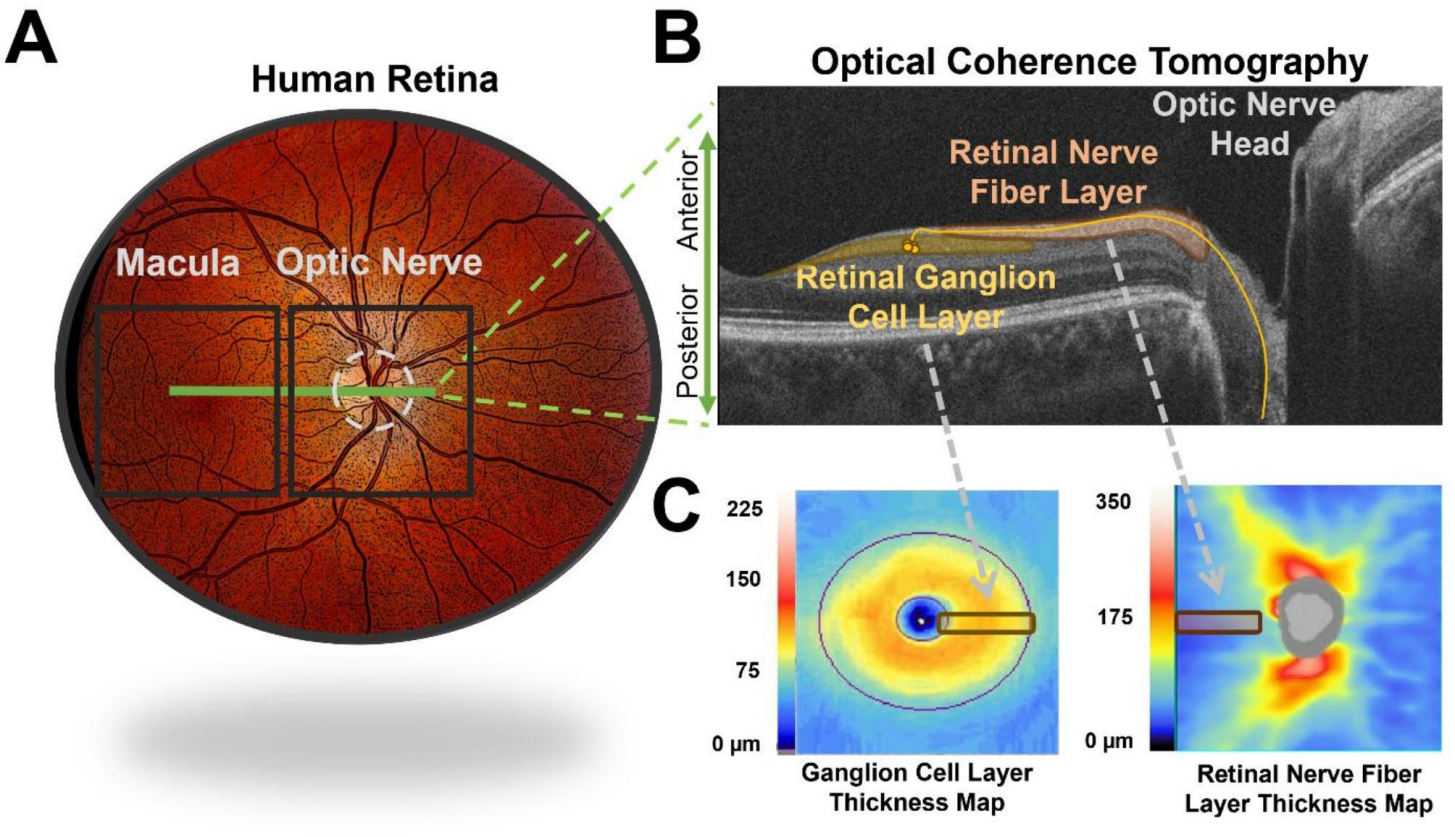
Optical coherence tomography (OCT) imaging of the optic nerve: **A**) Illustration of the posterior pole of the human retina, showing the area of typical clinical OCT images of the optic nerve head and macula (black boxes). **B**) High-definition raster image of a single OCT b-scan showing details of the retinal layers, highlighting the retinal ganglion cell (RGC) layer, where the ganglion cell bodies reside, as well as the retinal nerve fiber layer, where the RGC axons course toward the optic nerve head. The thickness of each of these layers is shown en face in panel C. **C**) The black boxes recapitulate the retinal area shown in panel B. Clinical reports use normative data to highlight areas of excessive thickening in cases of swelling, or thinning in cases of atrophy

Optical Coherence Tomography (OCT) has become an essential non-invasive method for evaluating retinal structures in patients with various eye diseases such as glaucoma, age-related macular degeneration, and diabetic retinopathy [23–25]. The ability of OCT to produce detailed images of the en face retina and the retinal layer’s architecture has significantly advanced the diagnosis and management of various eye conditions over time (Fig. 1).

**Fig. 2:**
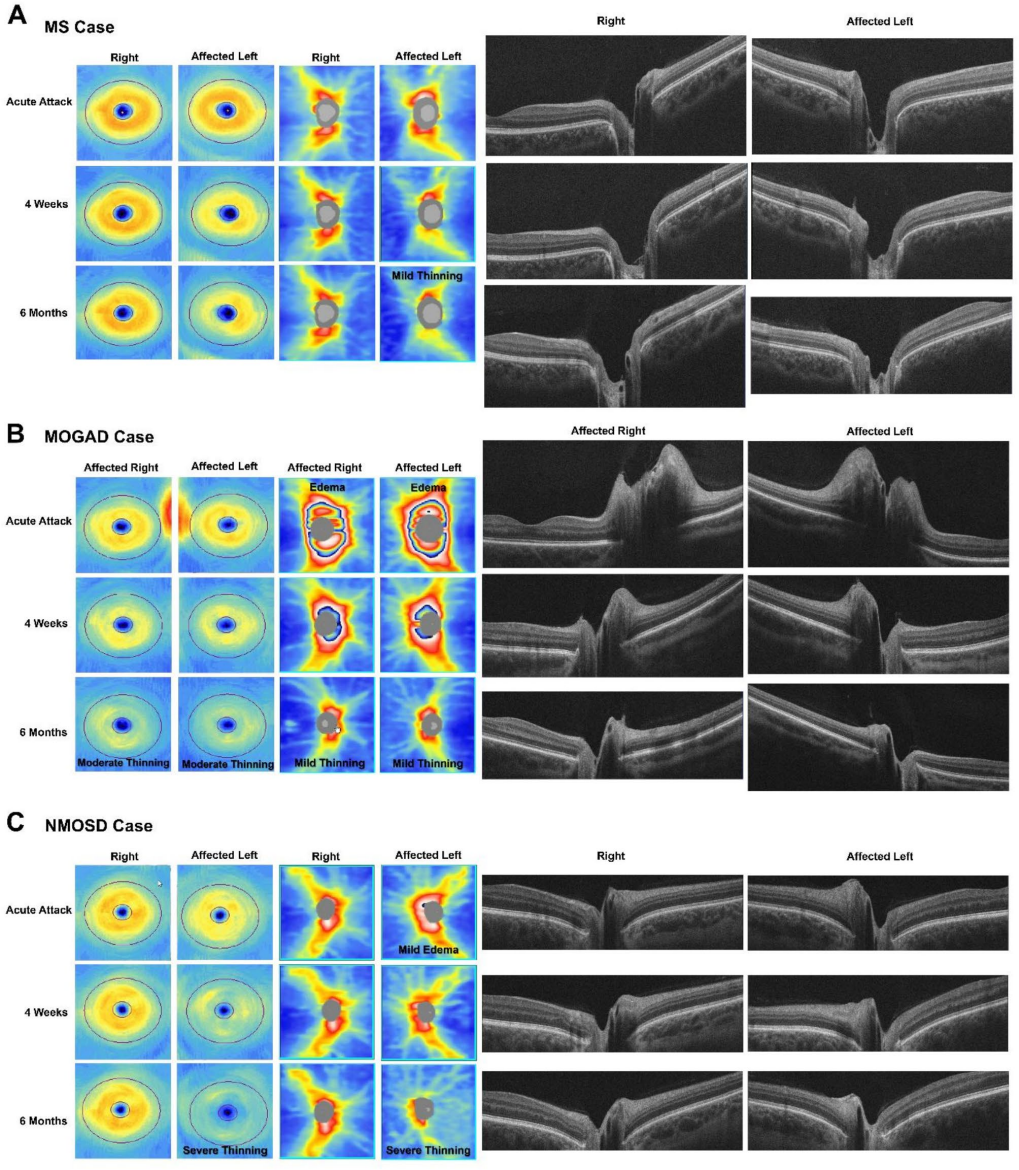
Optical coherence tomography images from prototypical cases of optic neuritis due to MS, MOGAD, and NMOSD. Each case shows a time series from the acute attack to follow up at 1 month and 6 months. Ganglion cell maps, retinal nerve fiber layer maps, and horizontal b-scans through the optic nerve are shown for each time point. **A**) In the MS case, there is minimal thickening at presentation, followed by moderate thinning over months. **B**) The MOGAD case shows significant optic disc edema in both eyes at the time of the acute attack, with progressive thinning to follow over months. **C**) In the NMOSD case, there is mild thickening at the optic nerve head during the acute attack, with early thinning of the ganglion cell complex evident at presentation. Following this, there is severe thinning of the RNFL and RGC complex that develops rapidly. Ganglion cell thinning is detectable earlier than RNFL thinning, typically within 6–10 days of onset. In humans, there is not typically any inner retinal edema from ON outside of the area just around the optic nerve, though microcysts can be seen in the outer plexiform layer beneath the ganglion cells in severe cases (not pictured here)

In cases of autoimmune demyelinating optic neuropathy, including ON, OCT imaging provides high-resolution, cross-sectional images of the retina for the precise measurement of the retinal nerve fiber layer (RNFL) and retinal ganglion cell (RGC) layer thickness [26]. Changes observed in the RNFL and RGC layers (as these translate to the optic nerve axons) are indicative of retrograde degeneration due to lesions in the optic nerve, chiasm, or optic tract. Notably, the thinning of the RNFL and RGC complex is linked to MS, where quantification of the RNFL reveals thinning over time, with a higher degree of thinning occurring in MOGAD-ON and NMOSD-ON when compared to MS-ON [22] (Fig. 2). Over the past decade, OCT imaging has grown in popularity in the ophthalmic and neurology fields where thinning of the inner retinal layers has been frequently used as secondary outcome measurements in clinical MS trials [27–29]. Degeneration of RGCs as a primary result of acute ON or secondarily through ongoing disease pathology such as incomplete resolution of central nervous system (CNS) inflammation, is also evident functionally in decreased pattern electroretinography (pERG) amplitudes [1, 27].

In addition to OCT imaging, OCT angiography (OCT-A) is widely used in clinical studies on MS and related inflammatory disorders. This non-invasive imaging technique allows clinicians to assess changes in retinal vasculature thought to be linked to underlying neurodegenerative processes [30]. Examination of patients with relapsing-remitting MS has demonstrated a characteristic thinning of the superficial vascular complex (SVC) with a predominant effect on small-sized vessels (diameter < 10 μm) irrespective of the patient’s ON history. Further involvement of medium-sized vessels (diameter 10–20 μm) has been shown to occur in those with a history of acute ON [30, 31]. While these alterations in retinal vasculature occur simultaneously to thinning of the common ganglion cell and inner plexiform layer (GCIPL), it is thought that vessel rarefication and RGC loss are independent findings [32–34]. Though not predictive of RGC loss, OCT-A remains a valuable tool to study MS-related neurodegeneration given the association between vessel loss and grey-white matter atrophy [33].

**Fig. 3:**
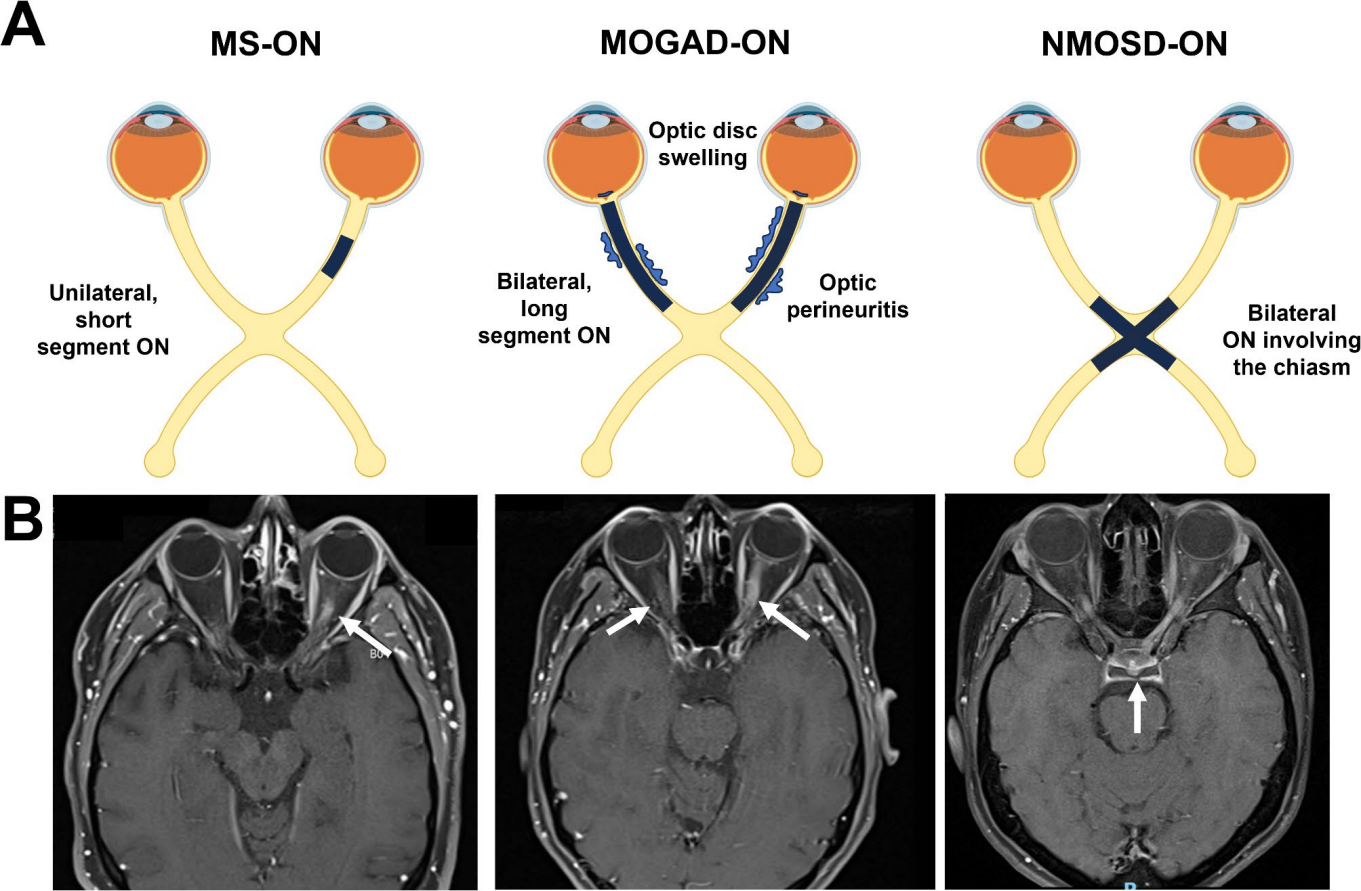
Representation of the optic nerve inflammation during acute phase optic neuritis in patients with MS, MOGAD and NMOSD. **A**) Schematic illustration of ON manifestations (dark blue areas) in MS involves unilateral short segment ON. Bilateral anterior ON in MOGAD is common and associated with accompanying optic disc edema extending more than 50% of optic nerve length bilaterally with optic nerve sheaths and perioptic fat involvement. AQP4 + NMOSD often represents bilateral ON involving the chiasm(Adapted from: Jeyakumar et al. Eye 2024, Cacciaguerra & Flanagan. Neurology Clinics 2024). Panel B) represents classic MRI findings in ON (arrows) due to MS which involves a short segment, often with patchy contrast enhancement in the optic nerve. In MOGAD, long-segment of contrast enhancement with involvement of the posterior globe is observed, and in NMOSD, a typical clinical manifestation is evident by bilateral involvement of the posterior optic nerves with involvement of the chiasm

Although the nature and frequency of changes in visual function and structure are indicative of demyelination disorders, a definitive diagnosis can often only be made upon subsequent neurologic diagnostics including magnetic resonance imaging (MRI) and serum antibody testing (in the case of MOGAD and NMOSD). According to the 2017 McDonald criteria for MS and the MAGNIMS consortium recommendations, MRI criteria for the diagnosis of MS are based on the presence of focal white matter lesions (T2-hyperintense or gadolinium-enhancing) and the demonstration of disease dissemination in both time and space [35, 36]. The MAGNIMS consortium further proposed to include presence of a lesion in the optic nerve as an indication of dissemination in space [36]. The proposed 2024 revision of the McDonald criteria includes the optic nerve as a classic diagnostic location for MRI changes in MS, in addition to other new MRI biomarkers. OCT measurements showing inter-eye differences of greater than 6 microns in the RNFL or greater than 4 microns in the GCIPL layer may also be included to substantiate a diagnosis of optic neuropathy [37, 38]. At the time of writing of this review, the updated criteria are not yet published. MRI diagnostics for MOGAD and NMOSD are more challenging because of the difference between the acute attack related phenotype and post-attack manifestations. For MOGAD, MRI findings are generally characterized by poorly demarcated tumefactive lesions in the subcortical white matter and deep grey matter. Longitudinally extensive lesions are also present centrally in the thoracic and lumbar spine [39, 40]. In addition to the presence of a MOGAD associated MRI phenotype, it is proposed that a correct diagnosis also relies on the detection of anti-MOG antibodies either in the serum or cerebrospinal fluid [4, 39]. When diagnosing NMOSD, characteristic MRI findings include dot-like lesions surrounding the third and fourth ventricles, as well as longitudinally extensive lesions in the spinal cord, corticospinal tract, and deep white matter. Additionally, the presence of serum aquaporin-4 (AQP4) IgG antibodies (AQP4-ab) can also be performed although not always present as a subgroup of patients have seronegative NMOSD [40]. Differences in the MRI phenotypes of MS, MOGAD, and NMOSD are also reflected in the patient’s optic nerves. The optic nerve MRI of MS patients typically indicate unilateral lesions of short segments along the intraorbital tract. In MOGAD, longitudinally extensive lesions usually span over half of the length of the optic nerve and involve the optic nerve sheath bilaterally [4, 41]. In NMOSD, longitudinally extensive lesions occur bilaterally at the posterior optic nerves and involves the optic chiasma [40] (Fig. 3).

### Pathobiology of MS, MODAD, and NMOSD and current treatments

The pathology of MS is generally characterized by the degeneration of oligodendrocytes leading to demyelination which is associated with a variable degree of axonal and neuronal loss. These degenerative processes are related to CNS inflammation that is composed by CD8-positive T lymphocytes and B-cells, microglia and macrophage activation, and astrogliosis. The rate of progression of MS is thought to be associated with incomplete resolution of neuroinflammatory processes and impairment of compensatory mechanisms [42, 43]. In contrast to MS, presence of MOG autoantibodies is the hallmark in MOGAD. These MOG autoantibodies and infiltration of CD4-positive T-cells are thought to drive MOGAD disease pathology. Activation of the complement cascade is found in a subset of MOGAD brain autopsy cases, suggesting a primarily autoantibody-mediated demyelination in MOGAD [41, 44]. In cases of seropositive NMOSD, the presence of AQP4-abs results in the production of interleukins in astrocytes, leading to downstream signaling that affects endothelial cells and disrupts their ability to maintain the blood-brain barrier (BBB), a protective layer of cells that line the surface of blood vessels in the brain. Once in the CNS, AQP4-abs target and bind to astrocytes, initiating a complement dependent cytotoxicity. The cascade leads to degranulation of polymorphonuclear (PMN) cells such as neutrophils and eosinophils and activation of natural killer (NK) cells which together lead to degeneration of astrocytes. The resulting astrocyte damage leads to the inability for the cell to support surrounding oligodendrocytes and neurons, resulting in a secondary demyelination [45]. These substantial differences in the pathophysiology of MS, MOGAD, and NMOSD may relate to different course and recovery of vision after ON.

While the underlying etiology of MS-ON, MOGAD-ON, and NMOSD-ON varies, there are similarities between their treatment. In cases of acute or subacute ON attacks, the first-line treatment is high-dose intravenous corticosteroids for MS, MOGAD, and NMOSD. Following intravenous administration, oral steroids are started with a gradual taper over weeks to months [19]. In cases of MS-ON, patients are often started on disease-modifying therapies (DMT) for long-term disease management. Commonly utilized DMT options include interferon beta, glatiramer acetate, and natalizumab. For NMOSD-ON, plasma exchange therapy (PLEX) performed early in the course of an acute attack has been associated with better visual outcomes, and PLEX is commonly used in severe cases of both NMOSD and MOGAD-ON [46–48]. Controversy still exists regarding the necessity and efficacy of this intervention, especially in MOGAD, MS, and idiopathic cases. For chronic disease control, intravenous immunoglobulin (IVIG) has been shown to prevent recurrence of NMOSD-ON and is started after a singular attack. The use of IVIG in MOGAD-ON is reserved for those with two or more attacks, but literature supporting its use is less established compared to NMOSD [2]. More recently, monoclonal antibody therapies have been developed for the treatment of seropositive NMOSD, showing significant reduction in the rate of relapse [49, 50]. The currently approved immunotherapies include eculizumab, ravulizumab, satralizumab, and inebilizumab [51–55]. While there are no standardized guidelines in place for the utilization of these medications, an established consensus was developed to guide individualized therapeutic decision-making [56]. Comparatively, there are no current monoclonal antibody therapies developed for the treatment of MOGAD. Despite this lack of immunotherapy, DMT can still be initiated for MOGAD patients with recurrent signs of disease. While the approach to DMT for MOGAD remains highly variable, there appears to be some agreement regarding the efficacy of rituximab, chronic IVIG, and anti-metabolite medications such as azathioprine and mycophenolate mofetil [57].

Despite current advances in pharmacological treatment of ON, long-term visual outcomes associated with the disease remain poor. Given the significant morbidity and mortality associated with MS, MOGAD, and NMOSD, further research is needed to gain insight into these autoimmune demyelinating diseases. Thus, animal models such as MOG_35 − 55_-induced EAE are invaluable tools to better understand the mechanism and efficacy of targeted therapeutics.

### In vivo modeling of optic neuritis

#### MOG_35-55_-induced EAE as a preclinical model of MS-like optic neuritis?

Experimental autoimmune encephalomyelitis is generally accepted as a useful preclinical model of MS and has been extensively explored to determine the immunological principles of inflammatory-mediated demyelination. Even though MOG_35 − 55_-induced EAE is primarily CD4-positive T-cell driven, whereas CD8-positive T-cells dominate the pathobiology found in MS patients [58], the MOG_35 − 55_-induced EAE model has significantly contributed to deciphering the basics of the complex immunologic and inflammatory mechanism leading to demyelination with subsequent neuronal loss. Furthermore, the MOG_35 − 55_-induced EAE model has been substantially utilized for the development of immunomodulatory drugs for MS; however, with regards to drug development, the model is not without controversy. While FDA-approved drugs such as beta-interferon, glatiramer acetate, and fingolimod have been successfully translated from MOG_35 − 55_-induced EAE studies to clinical use [59], several drawbacks are associated with the immunopathogenic differences between MOG_35 − 55_-induced EAE and MS. For example, preliminary evidence from MOG_35 − 55_-induced EAE studies demonstrated that anti-tumor necrosis factor antibodies effectively inhibit the development of the EAE phenotype by interfering with the lymphocytes during the effector phase of disease [60]. However, when utilized as a treatment for MS in a phase one clinical trial, participants experienced increased disease activity and immune activation confirmed by worsening lesions on gadolinium-enhanced MRI [61]. The use of CD28-specific monoclonal antibodies to upregulate regulatory T-cells in the MOG_35 − 55_-induced EAE model revealed dampening of proinflammatory cytokines, making it a promising treatment for neuroinflammatory conditions like MS. While these drugs were observed to have no toxic effect in mice, translation of CD28 antibodies to MS studies resulted in the development of life-threatening cytokine storm and subsequent multiorgan failure during the first-in-man study [62]. Because these adverse reactions are likely the result of immunologic inter-species differences, it may lead to the notion that MOG_35 − 55_-induced EAE is a misleading model of MS. However, it is important to remember that despite these translational failures, several well studied aspects of MS have been successfully studied in MOG_35 − 55_-induced EAE, such as gene susceptibility, immunoregulation, migration of immune cells, and nervous tissue destruction and repair, rendering it a useful model [63–65].

While no single animal model can fully recapitulate all aspects of human MS pathophysiology, the most-studied and utilized model is actively induced MOG_35 − 55_ EAE. In this model, self-antigens derived from CNS protein are introduced in susceptible animal strains to induce an autoimmune response [66]. While a variety of CNS proteins can serve as the self-antigen such as myelin basic protein (MBP) and proteolipid protein (PLP), the most readily used protein is MOG epitope 35–55 (MOG_35 − 55_). Because immunization with MOG_35 − 55_ alone is not sufficient to induce disease, it is combined with complete Freund’s adjuvant (CFA) which serves to activate mononuclear phagocytes and cytokine production [66, 67]. Animals are also injected with pertussis toxin, which increases the immune response and the permeability of the BBB and the blood-retina barrier (BRB), either alone or in conjunction with CFA allowing sufficient migration of myelin-specific T-lymphocytes to the CNS [58]. Once past the BBB, the lymphocytes are thought to be reactivated by both local and infiltrating antigen-presenting cells causing subsequent inflammation and activation of both monocytes and phagocytes. As a result of immune system activation in the MOG_35 − 55_-induced EAE model, animals typically experience an acute, monophasic, self-limited neuroinflammatory disease course [67, 68].

Given its artificial nature of induction, the MOG_35 − 55_ EAE model remains imperfect; nonetheless, it continues to be the most reliable in vivo model for ON in mice. This model has been extensively used to determine the immunological principles of immune mediated demyelination, and there is a common understanding that this model resembles certain aspects of neuroinflammatory diseases.

**Fig. 4:**
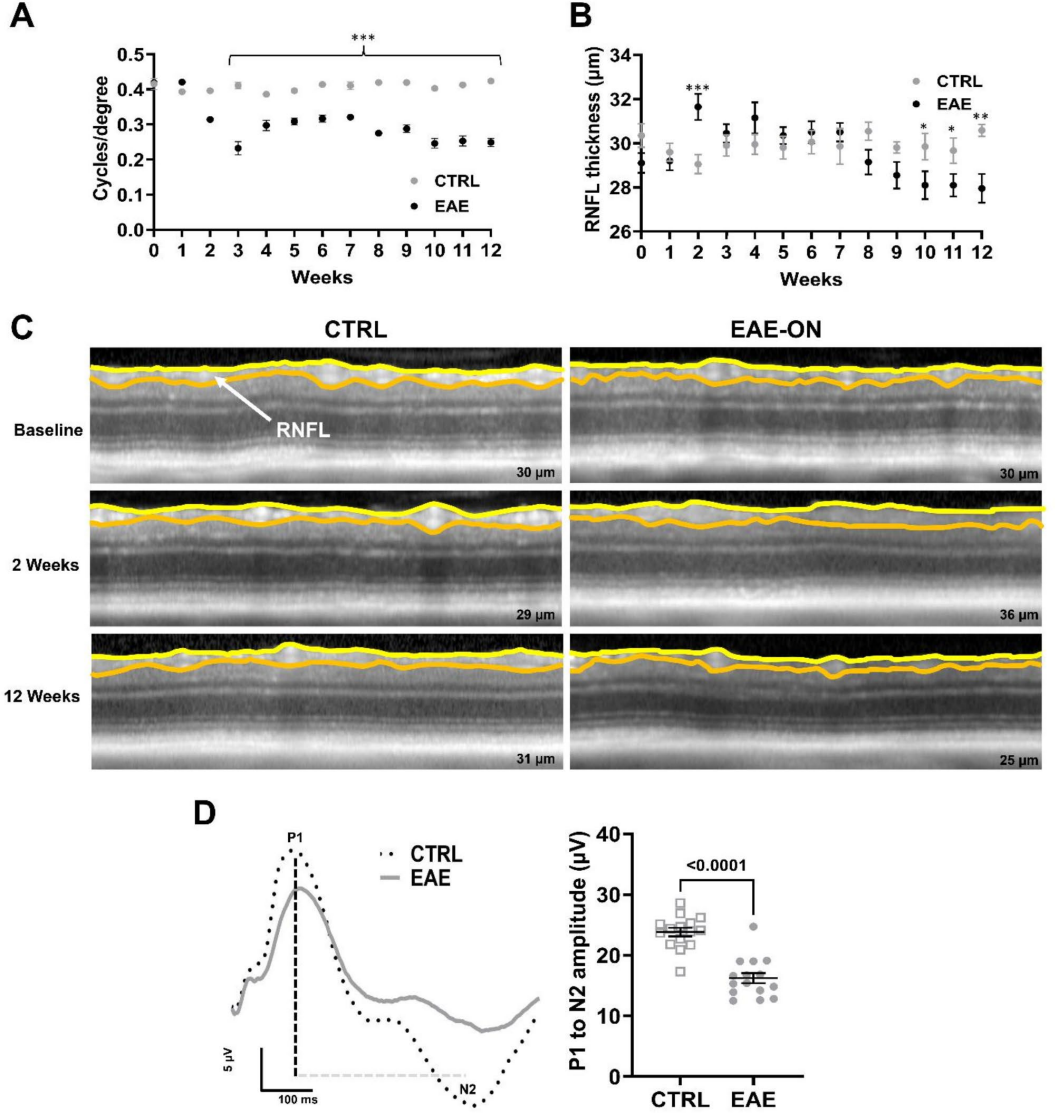
Time course of functional and structural changes within the visual system in EAE-induced mice. **A**) Visual acuity measured over time by changes in cycles/degree shows significant decline starting at two weeks post induction in MOG_35 − 55_-induced EAE mice compared to control mice, which is representative of other published studies (Godwin et al. Biomolecules 2022, Khan et al. Scientific Reports 2019). **B**) In accordance to Cruz-Herranz et al. Journal of neuroinflammation 2019 and Manogaran et al. Acta Neuropathologica 2019, analysis of average RNFL thickness over time demonstrates significant swelling followed by thinning of the RNFL in MOG_35 − 55_-induced EAE mice. For better transparency, methods relevant to EAE-induction and ophthalmic measurements shown in Fig. 4A and B are available in the Additional File 1. **C**) Representative OCT images measuring the RNFL indicate swelling during the acute phase of MOG_35 − 55_ EAE-ON and thinning at the end stage timepoint. **D**) Average pERG waveforms of MOG_35 − 55_-induced EAE and control mice show significant decrease in P1 to N2 amplitude in MOG_35 − 55_-induced EAE mice 60 days post induction (Fig. 4C and D adapted from Elwood et al. TVST 2024)

In particular, the MOG_35 − 55_-induced EAE model has served as a useful resource to study visual impairment in neuroinflammatory conditions because of its well-established ON phenotype. More importantly, the resultant ON in MOG_35 − 55_-induced EAE can be readily quantified using highly translational visual system matrices [69, 70] (Fig. 4). One such feature of the MOG_35 − 55_-induced EAE phenotype is decrease in visual acuity as commonly observed in ON patients assessed by high and low contrast visual acuity charts. However, because of the cognitive inability for rodents to participate in interactive visual acuity assessments, an indirect, non-conventional approach through the visual-vestibular pathway is required. In mice, the visual system uses image stabilization reflexes to compensate during fixation of moving stimuli. These reflexes result in compensatory head movement (optomotor response, OMR) along with compensatory eye movement (optokinetic response, OKR) and is a wieldy established measurement to determine visual system impairment in mice [71, 72]. By presenting MOG_35 − 55_-induced EAE animals with rotating fixation points of various widths, changes in visual acuity can be tracked throughout the disease course. Research examining the MOG_35 − 55_ EAE phenotype reveals acute worsening of visual acuity approximately 10–15 days after induction with slight recovery (or perseveration during treatment trials) over the following several weeks; however, there are no current reports indicating that visual acuity returns back to baseline [73–77].

Thinning of the retina is another feature observed in the MOG_35 − 55_-induced EAE visual phenotype and can be measured by OCT similarly to patients in the clinic. Following disease onset, the MOG_35 − 55_-induced EAE mice begin to have localized swelling of the RNFL and RGC layer. This thickening coincides with the onset of inflammation in both the retina and optic nerve [78]. Following the peak of disease severity, the edema resolves and is replaced by progressive retinal thinning. This gradual thinning can continue for months after disease onset even when stabilization of clinical symptoms has occurred [73].

Similar to the characteristic OCT findings, MOG_35 − 55_-induced EAE mice that have undergone genetic ablation of AQP4 display similar OCT-A characteristics to that seen in clinical studies of MS. Retinal involvement in these mice includes both a functional derangement of the retinal gliovascular unit with associated hyper-perfusion and a structural impairment of the BRB with extravasation of albumin during CNS inflammation. Ongoing inflammation in the retina leads to exaggerated upregulation of glial fibrillary acid protein which results in scarring and RGC loss [79].

In relation to retinal thinning observed on OCT, degeneration of RGC is seen in the MOG_35 − 55_ EAE phenotype which is quantified through retinal histological staining and fluorescent microscopy. The measurement of RGC density is a common biomarker used to study disease severity and the neuroprotective properties of proposed therapies. Loss of RGC occur as a result of inflammatory-mediated axon damage and begins approximately 15–30 days after EAE immunization [80, 81]. The degree of RGC loss in the MOG_35 − 55_-induced EAE model is suspected to be somewhere between 20 and 50% and is proportional to the severity of inflammation [82–85]. Further testing to assess RGC loss in the model is achieved using the electroretinogram, or pERG, which assesses the evoked electrical response of RGC from visual patterns such as black and white stripes varying in contrast and spatial frequency. In animals, the electrical activity of the retina can be assessed by stimulating the eyes with contrast-reversing visual stimuli. The measured output recorded through electrodes that are usually placed on the snout, eye (cornea), and head provide direct quantification of RGC dysfunction. The characteristic pERG finding seen in MOG_35 − 55_-induced EAE animals compared to controls is a decrease in the P1 to N2 amplitude, indicating less RGC cell stimulus evoked activity [70, 86, 87].

Another heavily studied and quantifiable visual system marker in the MOG_35 − 55_-induced EAE model is optic nerve histopathology. In the model, inflammation and cellular infiltration of the optic nerve results in loss of the myelin sheath protecting the axon. Histological evaluation of the MOG_35 − 55_-induced EAE model has shown that inflammatory cell infiltration occurs as soon as 9–12 days after immunization with subsequent demyelination over the next 1–2 days [82]. Through the use of myelin-specific staining, the extent of demyelination can be analyzed in the MOG_35 − 55_-induced EAE model [80]. Current histopathologic research demonstrates short segments of optic nerve demyelination in the MOG_35 − 55_-induced EAE model that spares the retrobulbar space. The demyelination is accompanied by axonal degeneration and infiltration of macrophages and T-cells [88, 89].

Because biopsy and histopathological analysis are not routinely performed for these diseases in humans, diagnostic methods such as MRI are used to characterize the location and extent of myelin damage. Despite these differences, research analyzing the ability of MRI to detect the histopathologic defects in the MOG_35 − 55_-induced EAE model show high sensitivity (100%) and specificity (90%), meaning that both are reliable methods to detect optic nerve demyelination [90]. Evaluation using both of these methods in MOG_35 − 55_-induced EAE animals have shown that demyelination most often occurs bilaterally in short segments and is located toward the middle region of the optic nerve with sparing of the immediately retrobulbar area [91, 92]. Similarly, axonal loss has been shown to occur in the MOG_35 − 55_-induced EAE model and is thought to result from prolonged inflammation and demyelination. Axon density can be assessed using histology and is an important outcome studied in research due to its correlation with permanent vision loss [82]. The demyelination and axon loss observed in the MOG_35 − 55_-induced EAE model can be further characterized by VEP. In these recordings, the electrical signal generated by the visual cortex in response to visual stimuli can be measured. Studies examining VEP in the MOG_35 − 55_-induced EAE model has shown that increased latency occurs early on in the disease course, followed by decreased amplitude later, coincident with axon loss [93].

When examining the MOG_35 − 55_-induced EAE model’s response to therapy, literature supports similar improvement between ON in the animal model and humans with MS-ON, MOGAD-ON, or NMOSD-ON. Examination of corticosteroid treatment in the MOG_35 − 55_-induced EAE model revealed suppression of ON and RGC loss when treatment was initiated prior to optic nerve inflammation. In animals treated after significant inflammation had begun, RGC loss was attenuated [94]. Additionally, research examining the effects of rituximab in the MOG_35 − 55_-induced EAE model revealed reduction in EAE clinical severity scores, impaired infiltration of T-cells into the perivascular space, and inhibition of inflammation and demyelination [95].

### Alternative and refined preclinical models of optic neuritis

While MOG_35 − 55_-induced EAE is one of the most commonly used preclinical models of MS-like ON, there are other models that resemble aspects of the neuroinflammatory phenotype. While these models vary in induction method and disease course, they all have the ability to provide unique insight into the understanding of MS, MOGAD, and NMOSD.

One alternative model uses PLP, a major protein of central nervous system myelin, to cause a relapse-remitting MS phenotype upon injection into SJL mice. Like the MOG_35 − 55_-induced EAE model, the PLP model utilizes CFA to induce active inflammation. However, PLP-induced EAE does not require the addition of pertussis toxin as in the MOG_35 − 55_ model. The encephalogenic portion of the PLP protein is 139–151 (PLP_139 − 151_) or 178–191 (PLP_178 − 191_) in the amino acid sequence. Previous investigators have found that 50nmol of PLP_139 − 151_ and 2 mg/ml *M. tuberculosis* is sufficient to induce severe EAE phenotype [96]. A major downside to utilizing SJL mice in any visual studies is that SJL mice are known to have a *Pde6b*^*rd1*^ deletion. This causes spontaneous degeneration of the rods within the retina and significant retinal thinning [97]. At this point in time, there are currently no reports characterizing the visual phenotype of the model and no means to backcross these mice to correct the *rd1* mutation [96].

Similar to PLP induction, SJL mice can also be actively induced with MBP, a molecule that plays an important role in the myelination of nerves. Induction can occur with full length MBP or at the epitope 84–104 (MBP_84 − 104_) in SJL or C57BL/6 mice. Like the MOG_35 − 55_-induced EAE model, MBP requires the addition of pertussis toxin to help the phenotype develop [95]. Unlike the PLP model, the MBP model requires 2 immunizations consisting of 100nmol of peptide that are performed 1 week apart. An advantage to the MBP model is that it can be induced in other rodents such as rats. Utilizing a similar induction scheme, MBP-induced EAE in Lewis rats causes an acute disease course that includes ON just like in mice [98].

The last form of active immunization for EAE utilizes human recombinant MOG protein. This can be induced in either SJL or C57BL mice using human MOG epitope 1-120 (MOG_1 − 120_). This contrasts the previously mentioned active induction methods due to the fact that human recombinant MOG is B-cell dependent thus reflecting the need for B-cell processing of the MOG protein for presentation whereas the other active inductions are more T-cell mediated disease [99, 100]. Both methods represent reliable models for testing therapeutic approaches. However, it has not been reported if human recombinant MOG_1 − 120_ produces an ON phenotype.

In addition to the active induction of EAE with PLP, MBP, or MOG, the EAE phenotype can be induced using a passive administration of activated lymphocytes. This passive method can be induced in either SJL or C57BL mice. Mice are first immunized with one of these encephalogenic peptides (PLP, MBP, or MOG). Around 10 days after the immunization, T-cells are isolated from the induced mice then subsequently activated with the respective peptide again. These immune cells can then be injected into naïve mice which causes EAE disease manifestation [96]. This method of passive EAE induction is noted to cause a more severe disease course along with accelerated disease progression. Additionally, the clinical and histological features of passive EAE induction by adoptive transfer are identical to that of active EAE induction with murine PLP, MBP, or MOG.

Alternative to active and passive induction, transgenic animal models are useful to study ON in mice that allow for an EAE phenotype without any protein immunization. In the T-cell receptor transgenic mouse (2D2^tg^), the phenotype results from transgenic modification that allows for the majority of CD4 T-cells to recognize a MOG antigen. As a result, a large proportion (> 30%) of mice spontaneously develop isolated ON without any clinical or histological evidence of EAE [101]. Further research examining the longitudinal progression of the model has displayed evidence of ON as well as spinal cord lesions on MRI. The lesions first develop at the optic chiasm and eventually spread to the spinal cord, presenting similarly to NMOSD. The 2D2^tg^ model provides beneficial insight on understanding AQP4-ab negative NMOSD that occurs in 10–40% of cases. Limitations to the model include the inability to reproduce some factors of neuroinflammation seen in other models of NMOSD [102].

Another transgenic animal model is the spontaneous opticospinal encephalomyelitis (OSE) model. The phenotype is the result of two transgenic modifications that cause the animal to express a T-cell rector capable of recognizing MOG_35 − 55_ peptide as well as B-cells with MOG-specific receptors. This allows the B-cells to function as antigen presenting cells to trigger disease onset through the activation of MOG-specific T-cells [103]. OSE animals have neuroinflammatory demyelination in the CNS, primarily affecting the optic nerves and lumbar spinal cord while sparing the brain and cerebellum. The ON that does occur tends to be bilateral and spans longitudinally through the entire section of the optic nerve [104]. Another OSE study reveals functional and structural retinal degeneration due to inflammation and complement activation leading to progressive RGC loss [105]. The spontaneous nature of the OSE model makes it ideal for studying environmental triggers. One drawback to this model is that EAE does not manifest in all animals, with studies showing approximately 50% of mice with the modifications forming the disease [103].

More recently, a refined MOG-immunization based model was described that characterizes both MOGAD and NMOSD more accurately to their respective clinical presentations. MOG-induced EAE was performed in C57B1/6JRj. At 10 days post induction, mice were given an intravenous injection of monoclonal murine anti-MOG IgG antibodies or purified human monoclonal recombinant anti-AQP4 IgG. MOG-IgG EAE animals had significant swelling within the ganglion cell complex during the acute phase which is a common clinical observation in MOGAD. After the acute phase, the ganglion cell complex thickness decreased significantly [106]. While the AQP4-IgG EAE animals did not show obvious difference in OCT measurements, the ganglion cell layer did swell slightly [107]. All animals had a decrease in visual acuity during both the acute phase and the chronic phase of the disease course. In MOG-IgG EAE animals, visual acuity significantly decreased during the acute phase, with a less pronounced decline during the chronic phase [106]. Conversely, AQP4-IgG EAE animals experienced a non-significant decrease in visual acuity in the early stage, but a more substantial decline in the chronic phase compared to the MOG-IgG EAE group [107]. These results align well with the visual acuity patterns observed in MOGAD and NMOSD patients, where both conditions lead to severe bilateral visual acuity loss, with NMOSD typically being more severe. In addition, the patterns of optic nerve histopathology in these animal models mirrors the respective clinical disease. MOG-IgG EAE animals display diffuse infiltration in the optic nerve across all disease states, whereas AQP4-IgG EAE animals show increased infiltration around the chiasm. These MOG-IgG EAE and AQP4-IgG EAE models recapitulate distinct aspects of MOGAD and NMOSD, respectively, and are potentially the closest in vivo model systems to the clinical phenotype yet [106, 107]. However, an important limitation to these models includes the limited availability of the disease-specific antibodies, especially the human recombinant AQP4-antibodies.

In the past decade, another NMOSD model has been described that does not require the administration of MOG antigen, a distinguishing feature from the one discussed above. The development of this model stems from the identification and mapping of the major T-cell epitope of AQP4, epitope 201–220 (AQP4_201 − 220_) [108]. While the natural T-cell repertoire is devoid of AQP4-specific T-cells in wild type mice, the use of a genetic AQP4 knockout mice (*Aqp4*^−/−^) allows for induction of a T-cell mediated response upon serum T-cell transfer or exposure to human-mouse chimeric recombinant AQP4-abs. While T-cell transfer alone has been shown to cause encephalomyelitis, a more robust phenotype mimicking NMOSD has been observed with the use of AQP4-abs or further transfer of immune serum from the *Aqp4*^−/−^ mice to Rag^−/−^ mice, an immunodeficient mouse model that lacks functional B-cells and T-cells [108, 109]. The resulting disease has been characterized by midline lesions in the brain, retinal pathology, and lesions at the grey-white matter border zone in the spinal cord [110].

### Translational characteristics and limitations of the MOG_35-55_-induced Eae model

#### Translational aspects of the MOG_35-55_-induced EAE model of optic neuritis

While all of the previously discussed animal models can serve as useful resources to further our understanding of neuroinflammatory disease, the MOG_35 − 55_-induced EAE phenotype remains especially useful due to its overlap with clinical manifestations of MS, MOGAD, and NMOSD.

In the clinic, patients with ON typically present with acute unilateral vision loss associated with painful eye movements and color desaturation. Visual acuity testing is performed using the Early Treatment Diabetic Retinopathy Study chart which often yields varied results depending on the skill of the examiner and the ability of the patient to successfully eccentrically fixate around a central scotoma. Despite differences in visual acuity, patients in the clinic almost invariably have some degree of acute visual field loss (97.5%), color vision disturbance (93.8%), decreased contrast sensitivity (98%), and a relative afferent pupillary defect [5]. In regard to vision loss observed in the MOG_35 − 55_-induced EAE model, the acute nature of ON and its associated visual acuity loss is similar to that of patients with MS, MOGAD, and NMOSD. As far as recovery goes, visual acuity returns close to baseline in MS patients with the majority starting to recover within the first month. This visual prognosis contrasts that seen in the MOG_35 − 55_-induced EAE model where only mild recovery is seen. This aspect of the MOG_35 − 55_ EAE phenotype is more translatable to MOGAD and NMOSD where mild to no recovery occurs with approximately 10% of MOGAD-ON cases and 25% of NMOSD-ON cases having long-lasting severe visual deficits [3, 19, 20, 111].

While histopathology is used commonly in MOG_35 − 55_-induced EAE studies as discussed above, its use in evaluation of human ON is only possible in post-mortem specimens, making for research that is limited in sample size. However, despite some variation between studies, current research has attempted to detail the extent and distribution of demyelinating plaques in patients with MS, NMOSD, and MOGAD [44, 112–115]. The histological patterns for individuals with MS tend to have more confluent areas of demyelination, with macrophage, B-cell, and T-cell infiltration at lesion borders and perivascular spaces, and loss of oligodendrocytes. In MOGAD, histological patterns tend to have multifocal perivenous inflammation and demyelination with preservation of oligodendrocytes, with B-cells, CD4 T-cells, and macrophages. The histopathology in NMOSD involves monocyte and T-cell infiltration with diffuse demyelination secondary to astrocytopathy, with loss of oligodendrocytes and RGC axon loss. Chronic inflammation outside of defined lesions has been associated with axonal degeneration in MS and in NMOSD, with little data reported in MOGAD [112–114]. Based on histopathology findings in the optic nerve, the MOG_35 − 55_-induced EAE model is most similar to MS in regard to demyelination pattern. However, there is a lack of B-cell infiltration in the MOG_35 − 55_-induced EAE model, which is different from MS and MOGAD, but similar to inflammatory infiltration seen in NMOSD.

While ON is a clinical diagnosis, the use of imaging can help confirm the diagnosis and stratify risk for disease development. Using standard MRI to evaluate the MOG_35 − 55_-induced EAE model, inflammation and demyelination has been shown to occur bilaterally in the anterior region of the optic nerve. This presentation is similar in location to MS where short retrobulbar segments can be found in the anterior segment of the optic nerve on MRI. The only slight variation between MS and the MOG_35 − 55_-induced EAE model is that MS presentation is unilateral in 95% of patients whereas the EAE model is mostly bilateral [1]. In terms of MOGAD and NMOSD, less similarities are seen between the human phenotypes and the model. MRI findings in MOGAD show bilateral demyelination that spans more than 50% of the anterior optic nerves, including the immediate retrobulbar area. Additionally, inflammation is seen at the optic nerve sheaths and perioptic fat. MRI findings for NMOSD reveal demyelination in the posterior portion of the optic nerve that extends into the optic chiasm, sometimes involving the optic tract [1, 19].

Another commonly used ancillary test used in the clinic to aid in the diagnosis of ON is OCT. Through this noninvasive imaging tool, both thickening and thinning of the retinal layers can be monitored. In addition to helping with diagnosis, OCT can be used to quantify thinning of the RNFL and GCIPL over years, which can be used as a biomarker of disease progression and impaired quality of life [116]. In the MOG_35 − 55_-induced EAE model, disc edema and thickening of the RNFL is seen early on in the disease followed by subsequent thinning of the RNFL and GCIPL [80]. In regard to RNFL thickening, the MOG_35 − 55_-induced EAE model closely resembles the course of MOGAD where moderate to severe disc edema is seen (85% of patients) and significant RNFL thickening occurs. In contrast, MS and NMOSD tend to have normal nerves or mild edema with slight thickening of the RNFL. A small proportion of patients have progressive thickening and microcystic edema in the inner nuclear layer of the retina, which is thought to be due to retrograde inner retinal degeneration, Müller cell dysfunction, or vitreous traction, and is not specific for demyelinating disease [117–119]. In regard to retinal thinning, all three neuroinflammatory diseases show thinning of the GCIPL layer, with NMOSD having the most profound loss [3].

While RGC density, axon loss, and demyelination are commonly quantified in EAE research studies using histology, these measurements can only be obtained post-mortem in patients with MS, MOGAD, and NMOSD as histological analysis cannot be performed in vivo. However, through the use of electrophysiology, information regarding the RGC and axon function can be assessed in the clinic. Similar to pERG techniques used on animals, electrodes can be placed on or around the eye. A visual patterned stimulus is then displayed, and the corresponding electrical signals evoked by the retina can be measured [120]. For VEP, electrodes are placed on the scalp in the occipital region and electrical activity of the cortex can be measured after administering visual stimuli [121]. While there is only limited electrophysiology data regarding MOGAD and NMOSD, findings on pERG and VEP in the MOG_35 − 55_-induced EAE model have been largely in agreement with data from human MS studies [93].

By assessing all these visual system markers together, it can be seen that the MOG_35 − 55_-induced EAE model does not fully resemble the entirety of MS, MOGAD, or NMOSD alone, but resembles aspects of all three conditions. More importantly, by exploring the translational value of the MOG_35 − 55_-induced EAE model to these conditions, we emphasize the importance of basic research in the development of new therapeutic applications and insights into these diseases. While many parallels can be made between the MOG_35 − 55_-induced EAE model and human disease, it is still important to keep in mind the differences that provide limitations on this model of ON.

### Shortcomings of MOG_35 − 55_-induced EAE model of optic neuritis

One such limitation is related to the phenotypical differences between humans and murine animals, such as mice and rats, which are commonly used in the MOG_35 − 55_-induced EAE model. These animals are non-foveal animals, meaning they do not have a true region of enhanced photoreceptor and retinal ganglion cell density where vision is primarily fixed. However, recent research examining murine retinas using population receptive-field mapping have identified a region of improved visual resolution located in the central-temporal retina of mice where receptive-fields of single-neurons are smaller [122]. In addition to this area of increased resolution, research looking at RGC loss in the MOG_35 − 55_-induced EAE model shows that majority of loss is seen in the mid-periphery, correlating to where a fovea would be in a human eye. Although not a perfect representation of the human retina, these pieces of information when taken together infer that therapies found to preserve retinal architecture in EAE animals may still be translatable to MS, MOGAD, and NMOSD.

Another limitation to the translation of experimental results from MOG_35 − 55_-induced EAE studies to the clinic are differences in the timeline of disease detection. Clinical signs in the MOG_35 − 55_-induced EAE model can be observed relatively soon after induction and proposed therapies can be implemented in the pre-clinical phase before symptoms even present. In comparison, diseases like MS, MOGAD, and NMOSD do not commonly have treatments started before clinical signs, meaning that therapies that display a prophylactic mechanism may not translate well from animal studies to humans [63]. Thus, the paradigm for drug testing using the MOG_35 − 55_-induced EAE model should consider appropriate timing, including interventional therapy regimens when motor or sensory symptoms first present as well as late-stage treatment when symptoms have already been established.

Phenotypical differences between the BBB and BRB in the MOG_35 − 55_-induced EAE model and neuroinflammatory diseases is another limitation in clinical translation. In the MOG_35 − 55_-induced EAE model, BBB and BRB disruption is induced through the use of pertussis toxin [63]. While similar to the disruption seen in MS, MOGAD, and NMOSD, the use of pertussis toxin in MOG_35 − 55_ EAE causes an artificial disruption that does not completely recapitulate the disruption seen in these diseases. Additionally, research has shown that MOG_35 − 55_-induced EAE animals continue to have a significantly disrupted BBB compared to human neuroinflammatory diseases where recovery and resealing of the BBB can occur. This provides a challenge in translating treatments that target the CNS as the therapeutic molecules may be able to migrate to these areas in the MOG_35 − 55_-induced EAE animals but not in patients with MS, MOGAD, and NMOSD [65, 123, 124]. Similarly to the BBB, disruption of the BRB in MOG_35 − 55_-induced EAE animals has been shown to occur early on in the disease with immunostaining revealing albumin accumulation around blood vessels in the retinal superior vascular plexus approximately 9 to 11 days post-induction [80, 125]. Compared to MOG_35 − 55_-induced EAE model, evaluation of the BRB in humans has shown the presence of microcytic edema, but disruption of the BRB is often unrelated to the clinical course with only a small percentage of patients with MS having complete blood ocular barrier breakdown [126, 127].

One final limitation within the MOG_35 − 55_-induced EAE animal model include the ability to measure OKR versus OMR to test visual acuity. OMR devices have emerged as a powerful tool to assess visual performance in mice but remain inferior to OKR measurements which are hard to implement and would require immobilization the animal’s head. In the MOG_35 − 55_-induced EAE model, where inflammation occurs in the spinal cord, it is possible that OMR measurements overestimate the degree of vision loss and that the animal is performing poorly because of inflammation in the spinal cord [71]. A more reliable assessment of visual acuity through the traditional cortical visual behavior pathway would be the object recognition swim test (Morris Water Maze) [128]. However, the forced performance of swimming in a paralyzed EAE mouse is highly questionable. Data from human studies indicate significant correlation between OKR and traditionally assessed visual acuity with eye charts [129], emphasizing the usefulness of nontraditional visual acuity measurement in EAE studies.

## Conclusion and future directions

Here, we present the immunologic and visual phenotypes of the widely used MOG_35 − 55_-induced EAE mouse model and alternative models. While primarily accepted as a preclinical model of MS, we argue that the unique pathophysiology and visual system findings actually characterize the MOG_35 − 55_-induced EAE model as primarily a MOGAD model with some aspects of MS and NMOSD (Table 1). In regards to pathophysiology, MOG_35 − 55_ EAE is induced through primarily a CD4-positive T-cell driven reaction, a similar mechanism seen in the development of MOGAD where anti-MOG antibodies cause CD4-positive T-cell infiltration that results in focal and confluent regions of demyelination [3]. This contrasts with MS pathophysiology which is thought to occur through inflammatory infiltrates composed of CD8-positive T-cells and B-cells [42]. Additionally, it differs from NMOSD which occurs from complement activation, infiltration, and degranulation of PMN cells, along with antibody-dependent cell-mediated cytotoxicity by NK cells [3].

In addition to pathophysiology, other features resembling the phenotype of MOGAD can be seen in the MOG_35 − 55_-induced EAE model. One such feature is visual acuity loss. In MOG_35 − 55_-induced EAE mice, subacute vision loss tends to be fairly moderate in nature and is present in the majority of mice. This parallels the severity and proportion of patients affected in MOGAD. Another finding in MOG_35 − 55_-induced EAE mice that resembles MOGAD is significant RNFL thickening seen at the beginning of disease development. The edema and thickening observed on OCT in the MOG_35 − 55_-induced EAE mice is similar to that seen in patients with MOGAD in the clinic. In contrast, these findings are notably mild or absent in MS and NMOSD patients. Lastly, the spine histopathology seen in MOG_35 − 55_-induced EAE mice resembles MRI findings in MOGAD which show long extensions of demyelination in the upper spinal cord and conus.

While the majority of the MOG_35 − 55_-induced EAE phenotype can be characterized under MOGAD, there are two important features to mention that more closely resemble MS and NMOSD. In regard to visual recovery, the MOG_35 − 55_-induced EAE model is most similar to NMOSD as poor recovery is observed. Furthermore, there is an incomplete recovery of visual function in the MOG_35 − 55_-induced EAE animals which differs from the almost complete recovery usually seen in the majority of MS and MOGAD patients. In contrast, the aspect of the MOG_35 − 55_-induced EAE model that does resemble MS is the location of the ON. The inflammation occurs unilaterally and is seen in short focal segments of the optic nerve which parallels the findings seen on MRI in MS patients.

**Table 1:** Comparison of the immunophysiology and visual phenotype of MOG_35 − 55_-induced EAE, MS, MOGAD, and NMOSD

	MOG_35 − 55_ EAE	MS	MOGAD	NMOSD
Pathophysiology	CD4-positive T-cells, complement activation	CD8-positive T-cells and B-cells, complement activation	CD4-positive T-cells, complement activation	PMN and NK cells, complement activation
Visual Acuity Nadir	Significant vision loss, visual acuity approximately half of baseline	Variable, often mild-moderate vision loss, ~ 35% with 20/200 or worse	Variable, often severe vision loss, ~ 70% with 20/200 or worse	Severe vision loss, ~ 85% worse than 20/200
Visual Recovery	Mild recovery, not back to baseline	Good, 95% with 20/40 or better	Good, but variable with 80% recover to 20/30 or better	Poor recovery, 50–70% with < 20/200 in at least one eye
MRI Ocular Findings	Unilateral or bilateral ON, short to moderate length segments of demyelination seen, spares retrobulbar space	Almost always unilateral ON, short segment enhancement of anterior optic nerve, spares retrobulbar space	Bilateral in 30–40% of cases, longitudinally extensive lesions of the anterior optic nerves, includes retrobulbar space	Bilateral in 20–30% of cases, enhancement at optic chiasm, sometimes involvement of posterior optic tracts
Histology	Short segments of demyelination, axonal degeneration, inflammation with macrophage and T-cell infiltration	Confluent areas of demyelination, inflammation present with macrophage, B-cell, and T-cell infiltrate at lesion borders/perivascular spaces, loss of oligodendrocytes	Multifocal perivenous inflammation and demyelination with B-cells, CD4 T-cells, and macrophages, preservation of oligodendrocytes	Monocyte and T-cell infiltration with diffuse demyelination secondary to astrocytopathy, loss of oligodendrocytes, RGC axon loss
MRI Spine Findings	Longitudinally extensive demyelination seen	Multiple short, focal areas of enhancement (peripheral white matter)	Longitudinally extensive myelitis in cervical and thoracic spine (gray matter only), conus involvement	Longitudinally extensive myelitis in cervical and thoracic spine (white and gray matter involvement)
OCT	Moderate RNFL thickening at day 14–21, followed by GCIPL thinning	Mild RNFL increase acutely, GCIPL thinning in following weeks	Significant RNFL thickening, early GCIPL loss	Variable RNFL thickening, profound GCIPL loss
Fundus	Moderate edema	Normal to mild optic disc edema (35%)	Moderate to severe disc edema (85%)	Variable, milder if present

Given the broad overlap of features from MS, MOGAD, and NMOSD, the MOG_35 − 55_-induced EAE animal model can serve as a comprehensive tool to further our understanding of visual impairment in all three of these autoimmune demyelinating conditions. It is positioned especially well for this role due to the translational ability of visual system biomarkers. The functional and structural markers described above, such as visual acuity, OCT, and electrophysiology, can be easily obtained from animals and humans acutely and over time to assess the natural history of the disease and treatment success. Given its translational value and unique phenotype, the MOG_35 − 55_-induced EAE model is as a critical resource to answer pressing gaps in our knowledge of ON.

Although no animal model of ON perfectly recapitulates human ON, they remain a valuable tool to gather insight into the clinical disease course and potential therapies. Through the careful assessment of the MOG_35 − 55_-induced EAE animal model, we put forth the idea that this model serves not only as research tool to study MS-ON, but that it offers a unique advantage to broaden our understanding of other neuroinflammatory conditions due to features that are observed in human ON due to MS, MOGAD, and NMOSD.

## Electronic supplementary material

Below is the link to the electronic supplementary material.

Supplementary Material 1

## Data Availability

Animal data generated or analysed during this study are included in this published article:” Elwood BW, Godwin CR, Anders JJ, Kardon RH, Gramlich OW. Correlation of Visual System Biomarkers With Motor Deficits in Experimental Autoimmune Encephalomyelitis-Optic Neuritis. Translational vision science & technology. 2024;13(8):1” and/or will be made available upon request.

## Abbreviations

AQP4: Aquaporin-4
AQP4-ab: Aquaporin-4 IgG antibody
Aqp4 ^−/−^: Aquaporin-4 knockout mice
BBB: Blood-brain barrier
BRB: Blood-retina barrier
CFA: Complete Freund’s adjuvant
CNS: Central nervous system
DMT: Disease-modifying therapy
EAE: Experimental autoimmune encephalomyelitis
GCIPL: Ganglion cell and inner plexiform layer
IVIG: Intravenous immunoglobulin
MBP: Myelin basic protein
MBP_84 − 104_: Myelin basic protein epitope 84–104
MOG: Myelin oligodendrocyte glycoprotein
MOG_35 − 55_: Myelin oligodendrocyte glycoprotein epitope 35–55
MOG: Myelin oligodendrocyte glycoprotein
MOGAD: Myelin oligodendrocyte glycoprotein antibody-associated disease
MRI: Magnetic resonance imaging
MS: Multiple sclerosis
NK: Natural killer
NMOSD: Neuromyelitis optica spectrum disorder
OCT: Optical coherence tomography
OCT-A: Optical coherence tomography angiography
OKR: Optokinetic response
OMR: Optomotor response
ON: Optic neuritis
OSE: Opticospinal encephalomyelitis
pERG: Pattern electroretinography
PLEX: Plasma exchange therapy
PLP: Proteolipid protein
PLP_131 − 151_: Proteolipid protein epitope 131–151
PLP_178 − 191_: Proteolipid protein epitope 178–191
PMN: Polymorphonuclear
RGC: Retinal ganglion cell
RNFL: Retinal nerve fiber layer
SVC: Superficial vascular complex
VEP: Visual evoked potential
2D2^tg^: T-cell receptor transgenic mouse
