# Supplementary material for: MOG35 − 55-induced EAE model of optic nerve inflammation compared to MS, MOGAD and NMOSD related subtypes of human optic neuritis
Source: J Neuroinflammation. 2025 Apr 7;22:102. doi: 10.1186/s12974-025-03424-4 (PMC11977933; doi:10.1186/s12974-025-03424-4)
Supplement: Supplementary file 1 — Supplementary Material 1 [file 12974_2025_3424_MOESM1_ESM.docx]

Additional file 1

All animal experiments were approved by the local IACUC and conducted according to the ARVO Statement for the Use of Animals in Ophthalmic and Vision Research. EAE-ON was induced in eight-to-twelve-week-old male and female C57BL/6J mice (The Jackson Laboratory, Bar Harbor, ME, USA). Briefly, mice were injected subcutaneously along the back with 250µg MOG_35-55_ (Sigma Aldrich, St. Louis, MO, USA) emulsified with complete Freund’s adjuvant (Sigma Aldrich) containing 2mg/ml of mycobacterium tuberculosis (BD Difco, Franklin Lakes, NJ, USA). Subsequently, mice were injected intraperitoneally with 400 ng of pertussis toxin (Sigma Aldrich) on day 0 and after 48 hours. Mice were housed in 12-hour light-dark cycle with food and water *ad libitum.* Motor-sensory impairment was monitored daily by a blinded and trained investigator utilizing a five point scoring system with the following criteria: 0 = no symptoms, 0.5 = partial tail paralysis, 1 = tail paralysis, 1.5 = partial tail paralysis and waddling gait, 2 = tail paralysis and waddling gait, 2.5 = partial limb paralysis, 3 = paralysis of one limb, 3.5 = paralysis of one limb and partial paralysis of another, 4 = paralysis of two limbs, 4.5 = moribund state, and 5 = death. Ten EAE-ON mice (five male, five female) and ten healthy controls (five male, five female) were euthanized each week for 12 weeks.

Visual acuity was measured weekly using an OptoDrum system (Striatech, Tubingen, Germany). Awake mice were placed in a closed chamber on a platform. The chamber displays a virtual cylinder projecting stripes of varying spatial frequencies at 99.8% contrast. Reflexive head movements were detected by automated software through a top-down camera. The highest spatial frequency as cycles/degree detected by each eye reflects visual acuity.

OCT imaging was performed in ketamine/xylazine anesthetized Mice as a baseline and endpoint measurement. Images were taken using a Spectralis SD-OCT imaging system (Heidelberg Engineering, Vista, CA, USA) coupled with a 25D lens for mouse ocular imaging (Heidelberg Engineering). Retinal nerve fiber layer thickness was measured using a glaucoma scan. Ganglion cell complex thickness was measured 2mm away from the center of the optic nerve head in the superior, inferior, nasal, and temporal quadrants.

Pattern ERG was conducted prior to euthanasia of each animal as an endpoint measurement on a JORVEC platform (Intelligent Hearing Systems, Miami, FL, USA). Animals were anesthetized with an intraperitoneal injection of ketamine/xylazine/acepromazine, and pupils were dilated with 1% tropicamide. The cornea was moistened with GenTeal.
